# A Case of Fungal Ball Caused by Aspergillus oryzae in a Sake Brewery Worker

**DOI:** 10.7759/cureus.95293

**Published:** 2025-10-24

**Authors:** Mieko Tokano, Tarumoto Norihito, Shigefumi Maesaki, Hiroshi Yamaguchi, Yu Hosokawa

**Affiliations:** 1 Department of Infectious Disease and Infection Control, Saitama Medical University, Moroyama, JPN; 2 Department of Microbiology, Saitama Medical University, Moroyama, JPN; 3 Department of Pathology, Saitama Medical University, Moroyama, JPN; 4 Department of Otorhinolaryngology, Saitama Medical University, Moroyama, JPN

**Keywords:** aspergillus oryzae, fungal ball, fungal sinusitis, koji, nihon-shu, sake

## Abstract

This is one of the rare, documented cases of non-invasive fungal sinusitis caused by *Aspergillus oryzae* in an immunocompetent worker exposed occupationally. A 34-year-old woman working at a *nihon-shu *(rice wine or sake) brewery presented to our hospital with a headache. The patient was diagnosed with a fungal ball and underwent endoscopic sinus surgery. As the condition was non-invasive, the patient recovered with surgery alone and did not require antifungal treatment. Although fungal growth was not observed in a sinus pus culture, a histopathological examination of the removed sinus tissue revealed *Aspergillus*-like hyphae. *A. oryzae* is widely used in the brewing of *nihon-shu*. A genetic analysis of the rRNA, β-tubulin, and aflatoxin-related genes identified the organism as *A. oryzae*. The patient also provided a sample of the *koji* (malted rice) routinely used in her workplace. The *koji* was cultured and analyzed, and the genetic sequences in all three regions were found to have a 100% match with the clinical specimen, strongly suggesting it as the source of infection. Furthermore, this strain was susceptible to antifungal agents. Although *A. oryzae *is usually considered non-pathogenic to immunocompetent humans, it should be considered as a possible causative organism in workers involved in sake brewing and related fermentation industries.

## Introduction

In December 2024, the traditional knowledge and skills of sake making, including *nihon-shu* (rice wine or sake), *shochu *(distilled liquor), *awamori *(distilled liquor), and *mirin *(sweet sake for seasoning), were inscribed on the United Nations Educational, Scientific, and Cultural Organization (UNESCO)’s Representative List of the Intangible Cultural Heritage of Humanity. Traditional knowledge and skills of sake making refer to the skills of *toji *(chief sake makers) and *kurabito *(sake brewery workers), who traditionally use *koji *(malted rice) to make sake, and these skills are representative of Japanese culture. The earliest record of sake production using *koji *dates back to the early eighth century [1].

*Aspergillus oryzae* is a filamentous fungus widely used in the brewing of *nihon-shu*, soy sauce, and miso [2]. During the *nihon-shu* brewing process, *A. oryzae* is sprinkled onto steamed rice to allow mold to grow and form *koji* [1]. Enzymes such as amylase and protease produced by* A. oryzae* break down starch and proteins in rice, generating sugars and amino acids that are necessary for fermentation [2].

*Aspergillus flavus* and *Aspergillus parasiticus* belong to the *Aspergillus* section Flavi along with *A. oryzae*, and are taxonomically very closely related. While some strains of *A. flavus* and* A. parasiticus *produce aflatoxins, *A. oryzae* has been proven to be non-aflatoxigenic [2].

*Aspergillus fumigatus* and *A. flavus* are the most common *Aspergillus *species that cause fungal sinusitis [3]. *A. fumigatus* typically causes non-invasive forms (fungal ball), while* A. flavus *is more often linked to invasive forms [4]. In invasive fungal sinusitis, antifungal therapy is administered in addition to surgery. In contrast, for non-invasive cases, surgery alone is usually sufficient, and in rare instances, conservative management may lead to improvement. Although *A. oryzae* is generally considered non-pathogenic to humans [4], we report a rare case of fungal sinusitis caused by *A. oryzae *in a female brewery worker engaged in *nihon-shu* production. This case highlights the potential occupational risk of *A. oryzae* infection.

## Case presentation

**Figure 1:**
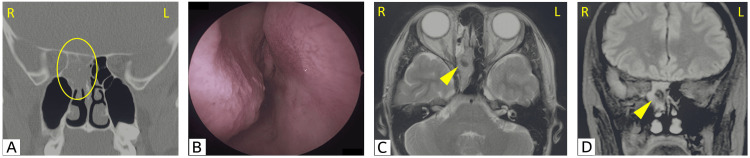
Findings on admission (A) Paranasal sinus CT image obtained at the initial visit. A soft tissue shadow with partial calcification was observed in the right sphenoid sinus and the right posterior ethmoid sinus (circle). No bone destruction was observed. (B) Nasal endoscopy findings at the initial visit. No purulent discharge or mucosal edema was observed. (C, D) Paranasal sinus MRI (C, T2-weighted image; D, FLAIR image) at the initial visit revealed a lesion in the right sphenoid sinus and the right posterior ethmoid sinus with low signal intensity (arrowheads). FLAIR: Fluid-attenuated inversion recovery

**Figure 2:**
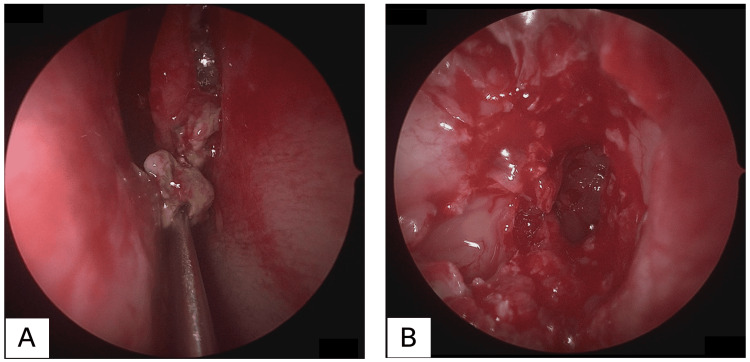
Findings during endoscopic sinus surgery (A) Upon opening the sinus, a yellowish lesion, suspected to be a fungal mass, was expelled from the natural ostium of the sphenoid sinus. (B) After the sinus was opened, the fungal mass was completely removed, and the sinus was thoroughly irrigated.

A 34-year-old woman presented to the emergency department with a one-month history of persistent headache. She was a brewery worker engaged in *nihon-shu* production and routinely handled *koji*. She had no underlying medical conditions. Despite taking loxoprofen prescribed at a nearby clinic, her bilateral headache did not improve. On presentation, her vital signs and chest and abdominal examinations were unremarkable, and no neurological abnormalities were observed. Head CT revealed no obvious intracranial abnormalities. However, a soft tissue shadow was observed in the right sphenoid sinus (Figure 1A), raising suspicion of sinusitis-induced headache. The patient was referred to our otolaryngology department. Nasal endoscopy revealed no purulent discharge or mucosal edema (Figure 1B). The lesion in the right sphenoid sinus showed low signal intensity on both T1- and T2-weighted MRI, suggesting a fungal ball. T2-weighted and fluid-attenuated inversion recovery (FLAIR) images are shown in Figures 1C-1D. Although her headache had improved by the time of her otolaryngology consultation, an invasive fungal ball could not be ruled out. Therefore, endoscopic sinus surgery was performed. Upon opening the sinus, a yellowish lesion, suspected to be a fungal mass, was expelled from the natural ostium of the sphenoid sinus (Figure 2A). After the sinus was opened, the fungal mass was completely removed, and the sinus was irrigated (Figure 2B).

Postoperative CT showed complete resolution of the soft tissue shadow in the right sphenoid sinus, and her symptoms improved. Histopathological examination revealed no mucosal invasion, and the diagnosis was non-invasive fungal sinusitis (fungal ball). No antifungal therapy was required. She returned to work, and no recurrence was observed during the 1.5-year follow-up period. From the perspective of antimicrobial stewardship, prophylactic administration is not recommended, and going forward, she was advised to visit a nearby medical facility if symptoms appear.

**Figure 3:**
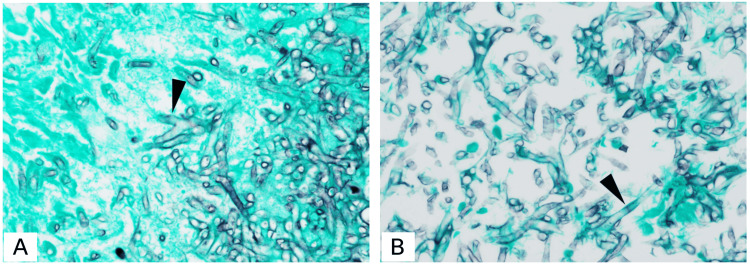
Histopathological images of the specimen excised during endoscopic sinus surgery (400× magnification) A tissue section of the excised specimen was prepared and stained with Grocott's methenamine silver, which revealed *Aspergillus*-like hyphae with Y-shaped acute-angle branching (A, arrowhead) and bamboo joint-like septa (B, arrowhead).

**Figure 4:**
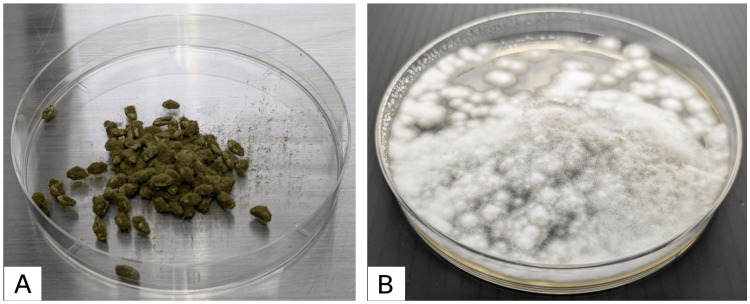
Koji and colony (A) The* koji *routinely used by the patient at her workplace. (B) The colony obtained by culturing the *koji* routinely used by the patient at her workplace at 35°C for 24 hours.

The specimen excised during surgery was cultured; however, fungal growth was not observed. A tissue section of the excised specimen was prepared and stained with Grocott's methenamine silver, which revealed *Aspergillus*-like hyphae with Y-shaped acute angle branching (Figure 3A) and bamboo joint-like septa (Figure 3B). To identify the fungus, nucleic acids were extracted from the excised specimen using a DNeasy Plant Mini Kit (QIAGEN, Venlo, Netherlands), and polymerase chain reaction (PCR) was performed. PCR and Sanger sequencing were conducted on three gene regions: rRNA, β-tubulin, and aflatoxin-related gene. For the rRNA and β-tubulin gene regions, *A. oryzae/flavus *was the only species that showed ≥99% identity in both regions. In the aflatoxin-related gene (*aflR*) region, *A. oryzae* RIB40 (Accession No: XM_023236965.1) showed 98.76% identity, while no other species showed ≥95% identity. In addition, all other aflatoxin-related genes besides *aflR* were negative [5]. Toxin production tests for* koji* are regularly conducted at her workplace, and all results have been negative. Based on these results, the organism was identified as *A. oryzae.* In addition, the patient provided a sample of the *koji *routinely used at her workplace (Figure 4A). The* koji* was cultured (Figure 4B) and analyzed. The sequences of all three regions matched 100% with those of the clinical specimen (Table 1) [5-8], strongly suggesting that it was the source of infection. Furthermore, antifungal susceptibility testing was performed in case medication would be needed if surgical treatment alone proved insufficient. The strain was susceptible to antifungal agents, as previously reported (Table 2) [9].

**Table 1:** Primer sequences used and results of the genetic analysis All other aflatoxin-related genes besides* aflR *were negative.

#Name	Reference	Genome size	Genome size obtained from the specimen	Genome size obtained from the *koji*	Sequence identity (%) between the specimen and the *koji*
ITS1 (TCCGTAGGTGAACCTGCGG) - ITS4 (TCCTCCGCTTATTGATATGC)	[6]	1237	1237	1237	100
NL1 (GCATATCAATAAGCGGAGGAAAA) - NL4 (GGTCCGTGTTTCAAGACGG)	[7]				
asap1 (CAGCGAGTACATCACCTTGG) - asap2 (CCATTGTTGAAAGTTTTAACTGATT)	[8]				
bT2a (GGTAACCAAATCGGTGCTGCTTTC) - bT2b (ACCCTCAGTGTAGTGACCCTTGGC)	[6]	545	136	545	100
aflR-F2 (CCGGCGCATAACACGTACTC) - aflR-R2 (GGCGCTTGGCCAATAGGTTC)	[5]	250	150	250	100

**Table 2:** Antifungal susceptibility of Aspergillus oryzae isolated from the cultured koji Antifungal susceptibility testing was performed according to CLSI document M38, 3rd Edition [10]. *Caspofungin was evaluated based on MEC, while the other antifungal agents were evaluated based on MIC. CLSI: Clinical and Laboratory Standards Institute; MIC: Minimum inhibitory concentration; MEC: Minimum effective concentration

Antifungal agent	Evaluation time (hour)	MIC/MEC (μg/mL)*
Caspofungin	24	0.25
Amphotericin B	48	0.5
Itraconazole	48	0.5
Voriconazole	48	0.25
Isavuconazole	48	0.5
Posaconazole	48	0.125

## Discussion

In fungal sinusitis caused by *Aspergillus* species, *A. fumigatus *is most commonly associated with non-invasive forms (fungal ball), whereas *A. flavus* is more frequently associated with invasive forms [4]. In cases of fungal sinusitis, although fungal elements may be confirmed in histopathological specimens, cultures often fail to yield growth, and the identification of species is difficult [11]. When culture is successful, morphological diagnostic methods are commonly used; however, in recent years, mass spectrometry (matrix-assisted laser desorption/ionization - time-of-flight mass spectrometry (MALDI-TOF MS)) has increasingly been employed. However, if the strain is not included in the database, there is a risk of misidentification as a closely related species. Therefore, molecular genetic techniques have become important supplementary tools for identifying fungal species. The commonly used genetic regions for fungal identification include the internal transcribed spacer (ITS) and the large subunit (LSU) D1/D2 domains of the rRNA gene. In addition, the β-tubulin and calmodulin genes were used for *Aspergillus* species identification. However*, A. oryzae* has many closely related species, which are generally difficult to identify using conventional fungal genetic markers [12]. In this case, based on previous reports [5,13], we analyzed the aflatoxin-related gene and found no other species with high sequence identity aside from *A. oryzae*. Moreover, the sequences of all three gene regions from the patient specimen matched 100% with those obtained from the* koji* routinely used in the patient’s workplace, indicating that the *koji* was *A. oryzae *and strongly suggested it to be the source of infection. Whole-genome sequencing could have been performed if fungal growth had been obtained from the sinus specimen; however, this was not possible because no fungal growth was observed.

Although* A. oryzae *is usually considered non-pathogenic to immunocompetent humans [4], only a small number of reported cases of fungal sinusitis suspected to involve *A. oryzae* exist (Table 3) [14,15]. However, none of these studies reported genome sequencing; therefore, misidentification cannot be completely ruled out. In addition, one study detected *A. oryzae* in one of 235 cases of maxillary sinusitis [16], and another study using next-generation sequencing identified *A. flavus*, *A. oryzae*, and *Myceliophthora thermophila *as the three major causative fungi in maxillary sinusitis [17]. These findings suggest that, in some cases previously diagnosed as* A. flavus* infections by conventional methods, *A. oryzae* may have been the causative organism. The patient in this case had no immunological abnormalities; however, she was continuously exposed to *A. oryzae* over a long period and likely inhaled its spores, which was considered the cause of infection.

**Table 3:** Reported cases of fungal sinusitis suspected to involve Aspergillus oryzae PCR: polymerase chain reaction; MALDI-TOF MS: matrix-assisted laser desorption/ionization - time-of-flight mass spectrometry; M: male; F: female

Age	Sex	Underlying disease	Identified species	Basis for identification	Treatment	Outcome	Reference
24 years	M	Acute promyelocytic leukemia	Aspergillus oryzae	Morphology of the cultured fungus	Surgical resection and intravenous administration of amphotericin B	Death	[14]
51 years	M	None. His occupation was in the fermentation industry (miso)	*Aspergillus *spp.	PCR of the cultured fungus (only electrophoresis performed, sequencing not conducted)	Endoscopic sinus surgery and local treatments, such as nasal irrigation with hydrogen peroxide	Recovered	[4]
76 years	M	Coronary arterial disease, spinous cell carcinoma	*Aspergillus flavus*/*oryzae*	Mass spectrometry (MALDI-TOF MS) using colonies of the cultured fungus	Endoscopic sinus surgery and intravenous administration of voriconazole at 6 mg/kg	Recovered	[15]
34 years	F	None. Her occupation was in the fermentation industry (sake)	Aspergillus oryzae	PCR using sinus specimens and Sanger sequencing of the PCR products	Endoscopic sinus surgery and local treatments, such as nasal irrigation with hydrogen peroxide	Recovered	Our case

Treatment of fungal sinusitis generally depends on the presence or absence of systemic symptoms. In cases with systemic involvement, such as pain or numbness due to trigeminal nerve impairment, oculomotor nerve palsy due to cavernous sinus invasion, or visual disturbances caused by optic canal involvement (i.e., invasive fungal sinusitis), antifungal agents are administered after surgical intervention. In the absence of such symptoms (i.e., in non-invasive fungal sinusitis), surgery alone is usually sufficient, and antifungal therapy is rarely required [18-21]. This treatment approach did not vary according to the* Aspergillus* species. Both *A. oryzae* and *A. flavus* are no exceptions. In the relevant literature (Table 3), non-invasive cases of fungal sinusitis involving *A. oryzae* typically improved without systemic antifungal treatment, whereas invasive cases required such treatment. However, as the differentiation between *A. oryzae* and *A. flavus *may not have been clear in earlier reports, some of the invasive cases may have been due to* A. flavus*. The present case involved non-invasive fungal sinusitis, which improved without systemic antifungal therapy. The strain isolated from the *koji* showed no evidence of antifungal resistance when compared to previous susceptibility reports.

## Conclusions

The patient in this case was a woman working in *nihon-shu* (sake) brewing who routinely handled *A. oryzae* as part of her job, suggesting constant exposure. Therefore, *A. oryzae* should be considered as a potential causative organism for individuals working in the brewing industry. Since all non-invasive forms of fungal sinusitis have the potential to progress to invasive disease, fungal sinusitis should always be considered in the differential diagnosis of patients presenting with headaches. Furthermore, in headache patients who work in the *nihon-shu* brewing industry, *A. oryzae*-associated fungal sinusitis should be considered. Workers in the brewing industry should take measures to minimize exposure to *koji *as much as possible. Although *A. oryzae* is generally considered to have low pathogenicity, it is difficult to distinguish it from *A. flavus*, which is commonly associated with invasive fungal sinusitis. Therefore, in patients with sinusitis who engage in* nihon-shu* brewing or related occupations, careful attention should be paid during the diagnosis and treatment.
